# Long-term exposure to residential greenness and neurodegenerative disease mortality among older adults: a 13-year follow-up cohort study

**DOI:** 10.1186/s12940-022-00863-x

**Published:** 2022-05-07

**Authors:** Lucía Rodriguez-Loureiro, Sylvie Gadeyne, Mariska Bauwelinck, Wouter Lefebvre, Charlotte Vanpoucke, Lidia Casas

**Affiliations:** 1 Interface Demography, Department of Sociology, Vrije Universiteit Brussel, Brussels, Belgium; 2 Flemish Institute for Technological Research (VITO), Mol, Belgium; 3 Belgian Interregional Environment Agency (IRCELINE), Brussels, Belgium; 4 Department of Family Medicine and Population Health, Social Epidemiology and Health Policy, University of Antwerp, Wilrijk, Belgium; 5 Institute for Environment and Sustainable Development (IMDO), University of Antwerp, Antwerp, Belgium

**Keywords:** Greenness, Mortality, Neurodegenerative disease, Dementia, Air pollution, Long-term exposure

## Abstract

**Background:**

Living in greener areas is associated with slower cognitive decline and reduced dementia risk among older adults, but the evidence with neurodegenerative disease mortality is scarce. We studied the association between residential surrounding greenness and neurodegenerative disease mortality in older adults.

**Methods:**

We used data from the 2001 Belgian census linked to mortality register data during 2001–2014. We included individuals aged 60 years or older and residing in the five largest Belgian urban areas at baseline (2001). Exposure to residential surrounding greenness was assessed using the 2006 Normalized Difference Vegetation Index (NDVI) within 500-m from residence. We considered all neurodegenerative diseases and four specific outcomes: Alzheimer’s disease, vascular dementia, unspecified dementia, and Parkinson’s disease. We fitted Cox proportional hazard models to obtain hazard ratios (HR) and 95% confidence intervals (CI) of the associations between one interquartile range (IQR) increment in surrounding greenness and neurodegenerative disease mortality outcomes, adjusted for census-based covariates. Furthermore, we evaluated the potential role of 2010 air pollution (PM_2.5_ and NO_2_) concentrations, and we explored effect modification by sociodemographic characteristics.

**Results:**

From 1,134,502 individuals included at baseline, 6.1% died from neurodegenerative diseases during follow-up. After full adjustment, one IQR (0.22) increment of surrounding greenness was associated with a 4–5% reduction in premature mortality from all neurodegenerative diseases, Alzheimer’s disease, vascular and unspecified dementia [e.g., for Alzheimer’s disease mortality: HR 0.95 (95%CI: 0.93, 0.98)]. No association was found with Parkinson’s disease mortality. Main associations remained for all neurodegenerative disease mortality when accounting for air pollution, but not for the majority of specific mortality outcomes. Associations were strongest in the lower educated and residents from most deprived neighbourhoods.

**Conclusions:**

Living near greener spaces may reduce the risk of neurodegenerative disease mortality among older adults, potentially independent from air pollution. Socioeconomically disadvantaged groups may experience the greatest beneficial effect.

**Supplementary Information:**

The online version contains supplementary material available at 10.1186/s12940-022-00863-x.

## Background

Worldwide, the population is ageing, driving a dramatic increase in the burden of neurodegenerative diseases [1]. Moreover, population ageing is occurring faster in urban areas [2], which could contribute to an exacerbated risk of neurodegenerative diseases following exposure to urban environmental hazards [3–5].

Exposure to green spaces may benefit urban dwellers by promoting healthy ageing [6] and reducing premature mortality [7]. Hypothesized mechanisms include inducing stress restoration, providing opportunities for physical activity and social cohesion, and mitigating environmental hazards [8]. However, the available evidence on the association between green spaces and neurodegenerative diseases among the older population is mixed and inconclusive, and studies have so far primarily focused on dementia or its precursor, cognitive decline [9–21]. As far as we are aware, only one ecological and three longitudinal studies investigated the relationship between exposure to green spaces and neurodegenerative disease mortality: two longitudinal studies reported no association [22, 23], while the other two observed a beneficial association [24, 25]. Of these, only two further used a specific outcome of neurodegenerative disease mortality, namely, dementia mortality [23, 24].

Among the suggested mechanisms underlying the beneficial effects of green spaces, mitigation of ambient air pollution may be important for neurodegenerative diseases, as it is estimated to account for 4% of the total risk of dementia in later life [26]. Still, the effect of air pollution on other neurodegenerative diseases remains unclear [27, 28].

Finally, the health-related effects of green spaces could differ across social strata, as individual and neighbourhood social factors may influence exposure and susceptibility to residential green spaces [29, 30]. However, this has been scarcely addressed in relation to neurodegenerative diseases [16, 21].

In this study we examined the association between long-term exposure to green spaces and general and specific neurodegenerative disease mortality among the older population (aged 60 years and older) residing in the five largest Belgian urban areas. Additionally, we assessed the potential role of air pollution, and effect modification by social factors (gender and individual and neighbourhood socioeconomic position) in the studied associations.

## Methods

### Study design and study population

We used a longitudinal dataset based on a linkage between the 2001 Belgian census (baseline) and register data on emigration and mortality for the follow-up period from October 1, 2001, until December 31, 2014. Furthermore, environmental data (i.e., green spaces and air pollution) were linked to this dataset using the residential address of each person at baseline. Our study population included all non-institutionalized individuals aged 60 years and older, and officially residing in one of the five largest Belgian urban areas (Antwerp, Ghent, Brussels, Charleroi, and Liège). These areas were identified following the definition of urban area provided by Statistics Belgium (in Dutch: *stadsgewest*), consisting of the city and its respective commuting zone [31]. Individuals with incomplete data on the residential address due to administrative inaccuracies were excluded (*n* = 11,824; 1.03%).

### Neurodegenerative disease mortality

Mortality data included the causes of death issued from the death certificates, using the codes from the 10th revision of the International Classification of Disease (ICD-10) [32]. We defined several outcomes of neurodegenerative disease mortality according to previous literature [23, 33]. We considered deaths from all neurodegenerative diseases [including dementia (ICD-10: F00-F03); motor-neuron disease (ICD-10: G12.2); Parkinson’s disease (PD) (ICD-10: G20-G22); Alzheimer’s disease (AD) (ICD-10: G30); other neurodegenerative diseases of the nervous system, not elsewhere classified (ICD-10: G31); and multiple sclerosis (ICD-10: G35)] and four specific outcomes: AD (G30); vascular dementia (F01); unspecified dementia (F03); and PD (G20-G22). Neurodegenerative diseases are often under-reported in death certificates [34, 35]. We therefore included all death certificates with any mention to the abovementioned outcomes (as an underlying, immediate, intermediate, or additional cause of death).

### Residential surrounding greenness

Surrounding greenness around the residential address at baseline was measured using the Normalized Difference Vegetation Index (NDVI). A detailed description of the methodology used for the obtention of the indicator, as well as on the individual assignment of the exposure can be found in Bauwelinck et al. (2021). In brief, this metric captures vegetation density and is derived by applying the ratio between visible and near-infrared light bands to atmospherically corrected satellite images [37]. These were obtained from the Landsat-5 for the period from May to September 2006, with 30 m resolution. A composite mosaic was retrieved combining greenest pixels to reflect the peak of the growing season (i.e., maximizing greenness) [38]. Negative NDVI values representing water surfaces were set to zero prior to the calculation of the residential surrounding greenness indicator [36]. The index therefore ranged from 0 to 1, corresponding to no green and maximum green density, respectively. We calculated the average index of surrounding greenness in three circular buffers at Euclidean distances of 300-m, 500-m, and 1000-m from the residence.

As an alternative indicator of exposure to green spaces, we used an aggregated measure at the census tract level on the percentage of households that reported very good provision of green spaces in their neighbourhood in the 2001 Belgian census. A detailed description of this exposure indicator can be found in Rodriguez-Loureiro et al. (2022) [39].

### Ambient air pollution

We considered the potential role of air pollution in the associations between residential surrounding greenness and neurodegenerative disease mortality (Fig. S1) [40]. Data was obtained from the Belgian Interregional Environmental Agency (IRCEL-CELINE) (www.irceline.be). Air pollutant concentrations are constantly measured through a vast network of monitoring stations and then used in spatial-temporal (kriging) interpolation models. These data are further combined with Gaussian models including traffic and industrial sources and meteorological data to estimate air pollutant concentrations at high spatial resolution (25 m) in the whole Belgian territory [41, 42]. We used 2010 annual average concentrations (μg/m^3^) of fine particulate matter (PM_2.5_) and nitrogen dioxide (NO_2_).

### Sociodemographic and socioeconomic characteristics

Sociodemographic and socioeconomic characteristics at baseline were obtained from the 2001 Belgian census. Sociodemographic covariates included age [both as a continuous and with a 5-year categorization, from [60–65) until [95 and older)], gender, migrant background origin [Belgium, other high-income country (HIC), and low and middle-income country (LMIC)] and household living arrangement [cohabiting, single and other (e.g., multigenerational households)]. Individual socioeconomic position (SEP) was measured through attained educational level (tertiary, higher secondary, lower secondary, and lower and no education) and housing tenure (owner and tenant). Neighbourhood SEP was approximated at the level of the census tract of residence, i.e., the smallest available geographical unit free from privacy restrictions. We obtained the census tract median net taxable household income in 2005, available from Statistics Belgium (https://statbel.fgov.be/en) only from that year onwards, and unemployment rate in the census tract derived from the 2001 Belgian census.

### Statistical analyses

We conducted multiple imputation to minimize potential selection bias introduced by missing information on any of the variables, observed in 19.6% of the study population. We used chained equations, carrying out 25 imputations with 10 iterations that generated 25 datasets. All the covariates were used in the process of multiple imputation. We further included as a predictor in the imputation process the Nelson-Aalen estimator of the cumulative hazard to the survival time for all neurodegenerative diseases [43].

We examined Spearman correlations between surrounding greenness, PM_2.5_ and NO_2_, and neighbourhood SEP. We specified Cox proportional hazards models using age as underlying time scale. Observations were censored at age of emigration from Belgium, age of death from other causes, or age at the end of follow-up. Included covariates were selected a priori based on previous studies [22, 24, 36] and on data availability. Our main model was built adjusting stepwise by covariates: Model 1 (M1) included the baseline hazard as a function of age with strata terms for each 5-year categorized age group and gender; Model 2 (M2) was further adjusted by migrant background, household living arrangement, educational level, and housing tenure; and Model 3 (M3) added the median household income of the census tract of residence. Additionally, all models included a frailty term to account for the cluster effects of residing in one of the five largest urban areas. Frailty terms are used to incorporate random effects in survival models according to the hierarchical structure of the data, which accounts for unmeasured effects on mortality of residing in each urban area and allows for different distributions of the underlying baseline hazard functions [44]. Results were expressed as Hazard Ratios (HR) and corresponding 95% confidence intervals (95% CI) of the associations between residential surrounding greenness and neurodegenerative disease mortality. The reported HRs were combined from the estimates obtained from the 25 imputed datasets according to Rubin’s rules [45]. We a priori selected a 500-m buffer from residence for surrounding greenness, following the methodology used in previous studies [36, 46].

To examine the linearity of the exposure-response relationship with surrounding greenness, we randomly selected one of the 25 imputed datasets and fitted natural splines with three degrees of freedom. We compared the improvement of goodness of fit with a likelihood ratio test (LRT) to the main model (M3). We observed deviations from linearity of the exposure-response associations for Alzheimer’s disease and unspecified dementia mortality. Graphical representations of the associations using natural splines with three degrees of freedom showed parabolic shapes, also for Parkinson’s disease mortality (Fig. S2). Hence, we used surrounding greenness both as a linear term and categorized into quintiles. For the linear term, mortality associations were reported by one interquartile range (IQR) increment in surrounding greenness.

In additional analyses we investigated the role of air pollution in the studied associations. We firstly further adjusted our main models (M3) for PM_2.5_ and NO_2_ concentrations. Next, we evaluated potential mediation by PM_2.5_ and NO_2_ in the associations between surrounding greenness and neurodegenerative disease mortality outcomes that were significant in our main models. We used the package mediation [47] in R statistical software to obtain the average causal mediation effects (ACME), also known as indirect effects, and the average direct effects (ADE) in each imputed dataset (*n* = 25). This allows to calculate the proportion of the total effect mediated by air pollution dividing the ACME by the total effect [ACME/(ACME + ADE)]. Here it can be interpreted as the proportion of risk reduction of neurodegenerative disease mortality after exposure to surrounding greenness explained by a reduction in PM_2.5_ or NO_2_ concentrations [48]. We computed the average of the estimates and the corresponding 95% Quasi Bayesian confidence intervals (95%CI), based on 1000 Monte Carlo simulations, obtained from the 25 imputed datasets.

To determine if the observed effect in our main models was modified by sociodemographic characteristics, we stratified our models by gender, educational level, and neighbourhood SEP (by quartiles of the median net taxable income in the census tract of residence), as previous literature has shown strongest associations in women and lower socioeconomic groups [16, 17, 21].

Finally, to assess the robustness of our findings, we conducted several sensitivity analyses of our main models: (1) using the 300-m and 1000-m buffer sizes of residential surrounding greenness; (2) using neurodegenerative disease mortality outcomes listed as the underlying cause of death as opposed to using any mention to these diseases in the death certificates; (3) using perceived neighbourhood greenness as an alternative indicator of exposure to green spaces; (4) adjusting the main models for the unemployment rate in the census tract as an alternative indicator of neighbourhood SEP; (5) limiting our analyses to specific population groups: (a) the complete case population (i.e., individuals with no missing data on the covariates); (b) non-movers, i.e., individuals who resided in the same census tract between 1991 and 2001 (10 years prior to baseline); (c) individuals who had at least 4.25 years of follow-up [i.e., who did not die or emigrated from the beginning of the study (1st of October 2001) until the year of measurement of the indicator of surrounding greenness (1st of January 2006)]; (d) individuals originating from Belgium; (e) individuals younger than 80 years old at baseline; and (f) individuals residing in the city, leaving out commuting zone residents.

All statistical analyses were conducted in R/4.0 [49], using the packages mice [50], survival [51], splines [49] and ggplot2 [52] and dependencies.

## Results

Our total (imputed) study population consisted of 1,134,502 individuals aged 60 years or older and residing in the five largest Belgian urban areas in 2001 (Table 1). During follow-up (2001–2014), 69,149 individuals (6.1%) died from neurodegenerative diseases as any cause of death, 21,039 (1.9%) died from Alzheimer’s disease (AD), and 31,302 (2.8%) died from unspecified dementia. Fewer individuals died from vascular dementia or Parkinson’s disease (PD) (0.5 and 0.9%, respectively). The average age at baseline was 72 years (SD ± 7.7). The population was mainly composed by women, residents originating from Belgium, individuals with primary or no formal education, and cohabiting with their partner. The median exposure to residential surrounding greenness (buffer size: 500-m) was 0.61 (IQR: 0.22). In the complete case population (*n* = 911,648), deaths from these diseases were slightly lower (e.g., death from all neurodegenerative diseases: *n* = 52,780, 5.8%), while environmental exposures were similar. A detailed description of all environmental variables in both populations is shown in Table S1.

**Table 1:** Neurodegenerative disease mortality (2001–2014) and baseline characteristics in the full imputed and complete case population

	Full (imputed) population (***n*** = 1,134,502)	Complete case population (***n*** = 911,648)
**Neurodegenerative disease mortality, n (%)**		
All neurodegenerative diseases	69,149 (6.10)	52,780 (5.79)
Alzheimer’s disease	21,039 (1.85)	16,161 (1.77)
Vascular dementia	5,651 (0.50)	4,204 (0.46)
Unspecified dementia	31,302 (2.76)	23,570 (2.59)
Parkinson’s disease	10,054 (0.89)	7,932 (0.87)
**Age at baseline, mean ± SD**	71.8 **±** 7.7	71.5 ± 7.6
**Women, n (%)**	644,687 (56.8)	514,121 (56.4)
**Migrant background, n (%)**		
Belgian	1,001,938 (88.3)	813,136 (89.2)
Other HIC	94,811 (8.4)	70,497 (7.7)
LMIC	37,753 (3.3)	28,015 (3.1)
**Educational level, n (%)**		
Tertiary	143,927 (12.7)	118,543 (13.0)
Higher Secondary	182,750 (16.1)	150,398 (16.5)
Lower Secondary	299,778 (26.4)	242,632 (26.6)
Low/Primary	508,047 (44.8)	400,075 (43.9)
**Housing tenure, n (%)**		
Owner	859,624 (75.8)	700,281 (76.8)
Tenant	274,878 (24.2)	211,367 (23.2)
**Household living arrangement, n (%)**		
Cohabiting	715,546 (63.1)	591,646 (64.9)
Single	389,895 (34.4)	298,746 (32.8)
Other	29,061 (2.6)	21,256 (2.3)
**Median net taxable income (€), median (IQR)**	19,094 (4620)	19,206 (4530)
**Non-movers (1991–2001), n (%)**	1,003,052 (88.4)	810,682 (88.9)
**Urban area, n (%)**		
Antwerp	248,391 (21.9)	207,726 (22.8)
Ghent	120,809 (10.6)	101,904 (11.2)
Brussels	505,297 (44.5)	403,091 (44.2)
Charleroi	104,774 (9.2)	78,782 (8.6)
Liège	155,231 (13.7)	120,145 (13.2)
**Residential surrounding greenness (NDVI, 500-m), median (IQR)**	0.61 (0.22)	0.61 (0.22)
**Ambient air pollution**		
**PM**_**2.5**_ **(μg/m**^**3**^**), median (IQR)**	19.05 (2.09)	19.07 (2.09)
**NO**_**2**_ **(μg/m**^**3**^**), median (IQR)**	26.56 (12.22)	26.77 (11.98)

Note: *SD* Standard Deviation, *IQR* interquartile range, *€* euro currency, *NDVI* Normalized Difference Vegetation Index, *PM*_2.5_ Particulate Matter with an aerodynamic diameter < 2.5 μm, *NO*_2_ Nitrogen dioxide

Five largest Belgian urban areas, 2001–2014

Spearman correlations are displayed in Fig. S3. We observed moderate to strong negative correlations between surrounding greenness and air pollution concentrations (e.g., for surrounding greenness within 500-m and PM_2.5_: *r* = − 0.65). Neighbourhood SEP (median income) was moderately correlated with surrounding greenness (*r* = 0.50 for all buffer sizes), and weakly negatively correlated with air pollution (e.g., with PM_2.5_: *r* = − 0.1).

**Fig. 1:**
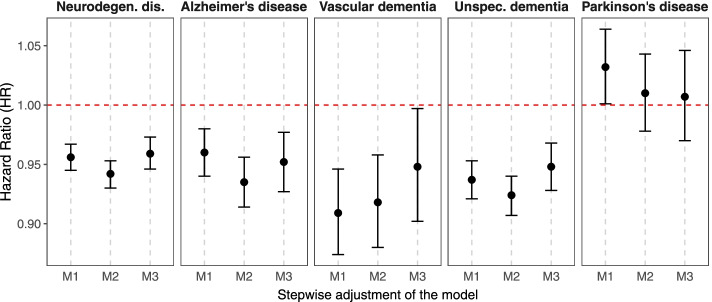
Associations (HRs) and 95%CI between residential surrounding greenness and *all* and specific neurodegenerative disease mortality. Legend: Five largest Belgian urban areas, 2001–2014. Results from Cox regression models using age as the underlying time scale. Model 1 included the baseline hazard, the strata terms for age and gender, and a frailty term for the urban areas; Model 2 added migrant background, household composition, educational level, and housing tenure; and Model 3 added median net taxable income at the statistical sector level. HRs expressed as one IQR increment (0.22) of residential surrounding greenness (buffer size 500-m) and neurodegenerative disease mortality outcomes

Surrounding greenness associations generally attenuated with increasing covariate adjustment in our stepwise models. Associations in fully adjusted models (M3) showed a reduction of 4% in the risk of all neurodegenerative disease mortality, and of 5% in the risk of AD, vascular dementia, and unspecified dementia mortality (Fig. 1 and Table S2). Contrarily, for PD mortality, we observed a non-significant, almost null association. Using the surrounding greenness indicator categorized into quintiles we observed the strongest associations for all neurodegenerative disease outcomes for the greenest quintile compared to the least quintile (Table S3). For AD mortality, we observed beneficial significant associations only for the greenest quintiles (Q4 and Q5) compared to the first quintile (Q1) (e.g., for Q5: HR 0.879, 95%CI: 0.834, 0.927). For PD mortality, we found inverse estimates (i.e., HR < 1) in all quintiles, but not significant, and the strongest effect in the third quintile, Q3, relative to the first (HR 0.960, 95%CI: 0.897, 1.027).

When further adjusting our main models for air pollution concentrations (PM_2.5_ and NO_2_) we generally observed an attenuation in the association between surrounding greenness and all neurodegenerative disease mortality (Table 2). Associations became null for AD and vascular dementia mortality and became stronger for unspecified dementia mortality. Results from the mediation analyses by PM_2.5_ and NO_2_ are reported in Table S4. Assuming that all statistical assumptions to conduct mediation were met [53], our findings suggest that the average proportion of the effect of surrounding greenness on all neurodegenerative disease mortality mediated by NO_2_ concentrations was 0.39 (95%CI: 0.09, 0.76). Results for PM_2.5_ were similar, but not significant. Furthermore, findings were suggestive of potential mediation in the association between greenness and vascular dementia mortality by NO_2_ (proportion mediated: 0.73, 95%CI: − 0.35,4.26). Evidence of mediation for other causes of death or PM_2.5_ was weak given observed opposite signs between ACMEs and ADEs.

**Table 2:** Associations between surrounding greenness and neurodegenerative disease mortality outcomes after adjustment for air pollution concentrations

	Neurodegene-rative disease mortality	Alzheimer’s disease mortality	Vascular dementia mortality	Unspecified dementia mortality	Parkinson’s disease mortality
	HR (95%CI)	HR (95%CI)	HR (95%CI)	HR (95%CI)	HR (95%CI)
**Main model (M3)**	0.959 (0.946, 0.973)	0.952 (0.928, 0.977)	0.949 (0.903, 0.998)	0.948 (0.928, 0.968)	1.007 (0.970, 1.046)
**Main model (M3) + PM**_**2.5**_ **(μg/m**^**3**^**)**	0.966 (0.948, 0.985)	1.004 (0.969, 1.040)	1.014 (0.948, 1.084)	0.918 (0.892, 0.944)	0.992 (0.943, 1.044)
**Main model (M3) + NO**_**2**_ **(μg/m**^**3**^**)**	0.972 (0.953, 0.991)	1.023 (0.987, 1.060)	0.986 (0.922, 1.055)	0.918 (0.892, 0.945)	1.014 (0.963, 1.068)

*Abbreviations: HR* Hazard Ratio, *95%CI* 95% confidence intervals, *PM*_2.5_ particulate matter with an aerodynamic diameter < 2.5 μm, *NO*_2_ nitrogen dioxide, μg/m^3^ micrograms per cubic metre

Five largest Belgian urban areas, 2001–2014

Cox regression models using age as the underlying time scale for the follow-up period October 1, 2001 - December 31, 2014. Main model (M3) stratified by gender and 5-year age groups, adjusted by migrant background, household living arrangement, educational level, housing tenure, median net taxable income at the level of the statistical sector, and including a frailty term for the urban areas

**Fig. 2:**
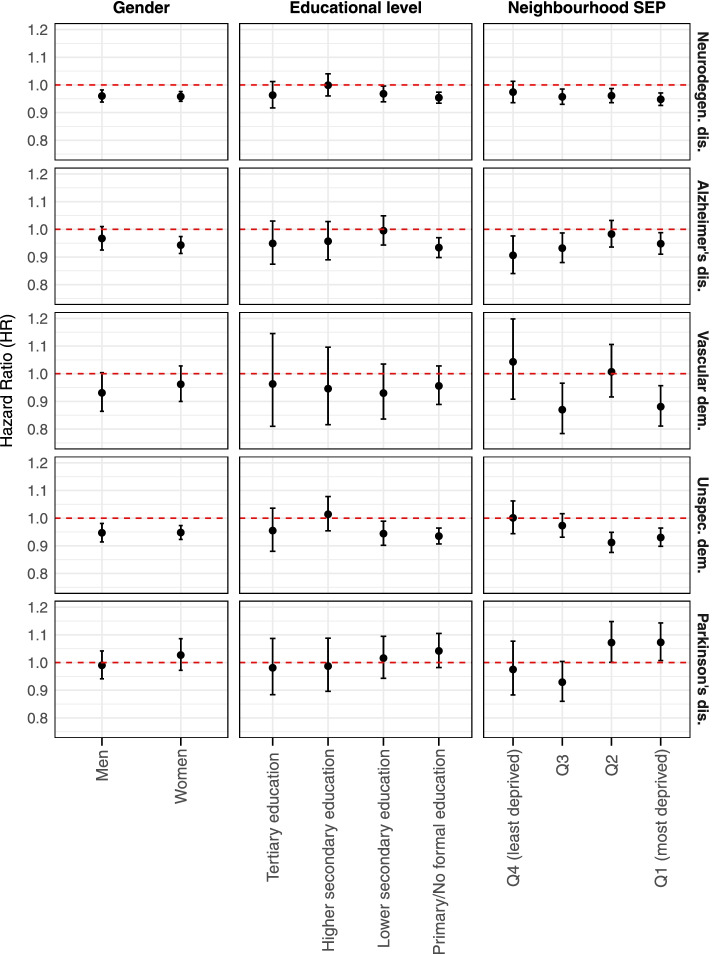
Associations (surrounding greenness and neurodegenerative disease mortality) stratified by gender, educational level, and neighbourhood SEP. Legend: Five largest Belgian urban areas, 2001–2014. Results from Cox regression models using age as the underlying time scale. Models included strata terms for age and gender, a frailty term for urban areas, and were adjusted for migrant background, household composition, educational level, housing tenure, and median net taxable income at the census tract level. Quartiles of exposure of area median net taxable income: Q1 (5676-16,471], Q2 (16,471–19,094], Q3 (19,094-21,091], Q4 (21,091-51,473]

The results of the stratified analyses by gender, educational level, and neighbourhood SEP are shown in Fig. 2 and are fully reported in Table S5. All subgroup effect estimates presented overlapping confidence intervals. We found almost no differences between men and women. We did not observe a clear pattern across educational levels. However, individuals with primary or no formal education showed consistently a stronger beneficial effect of surrounding greenness for all neurodegenerative disease, AD, and unspecified dementia mortality [e.g., for AD: HR 0.93 (95%CI: 0.90, 0.97)]. By neighbourhood SEP, we observed that for all neurodegenerative disease and unspecified dementia mortality the beneficial effect of surrounding greenness was strongest in individuals living in the most deprived (Q1 and Q2) neighbourhoods [e.g., for unspecified dementia: HR 0.93 (95%CI: 0.90, 0.96), HR 0.91 (95%CI: 0.88, 0.96); respectively]. Contrarily, for AD mortality this was observed in residents from the least deprived (Q4) neighbourhoods [HR 0.91 (95%CI: 0.84, 0.98)]. For vascular dementia mortality significant beneficial associations were observed in individuals residing in the second least deprived (Q3) and the most deprived (Q1) neighbourhoods.

The results of the sensitivity analyses were overall consistent with our main findings shown in Fig. 1 (Table S6-S7). The results using different buffer sizes were very similar. Results using neurodegenerative disease mortality outcomes listed as the underlying cause of death in death certificates were generally in line with our main models, although weaker for all neurodegenerative disease and AD mortality, and slightly stronger for vascular and unspecified dementia disease mortality. Using perceived neighbourhood greenness as alternative exposure indicator yielded null associations with AD and vascular dementia mortality, whereas with unspecified dementia it was stronger (HR 0.93, 95%CI: 0.91, 0.94). The associations with AD mortality lost significance after alternatively adjusting for unemployment rate in the census tract; in contrast, the inverse associations with vascular and unspecified dementia mortality were stronger. Using the complete case population and including only the population groups aged 60–80 and living in the city centre attenuated the associations, yielding in some cases a borderline non-significant association. Limiting the analyses to non-movers and residents originating from Belgium strengthened the observed associations, whereas for individuals who did not die or emigrate until exposure measurement (2006) the results were very similar but slightly attenuated for vascular dementia mortality. Finally, we observed an inverse but non-significant association with PD mortality when excluding individuals living in the commuting zone (HR 0.96, 95%CI: 0.91, 1.02).

## Discussion

Our findings suggest that exposure to urban residential greenness reduces mortality from all neurodegenerative diseases, and specifically from Alzheimer’s disease (AD) and dementia. We found no significant associations with Parkinson’s disease (PD) mortality. Associations remained robust for all neurodegenerative disease mortality when accounting for air pollution, but not for most specific mortality outcomes. Moreover, we found that this protective effect was generally stronger in individuals with lower education. We also found the strongest beneficial associations for all neurodegenerative diseases and unspecified dementia mortality in individuals residing in the most deprived neighbourhoods. In contrast, for AD mortality, the strongest beneficial association was observed in wealthier neighbourhoods.

Our results regarding reduced premature mortality from all neurodegenerative diseases with increased surrounding greenness are comparable to those reported by Klompmaker et al. (2021) in a study over 10 million adults (aged ≥30 years). Using the same classification of neurodegenerative diseases, the authors observed a 2% reduction (per 0.14 increase) in neurodegenerative disease mortality with surrounding greenness. Stratified by age, results were similar in older adults (≥65 years) [24]. An ecological study in Greece also found a significant inverse association (HR 0.91) between greenness and mortality from diseases of the nervous system (ICD-10 codes G00-G99) [25]. Two longitudinal studies found a protective but non-significant association with neurodegenerative disease mortality [22, 23]. James et al. (2016) included 108,630 older female nurses followed between 2000 and 2008, yielding an HR of 0.93 per 0.1 increase in surrounding greenness. Klompmaker et al. (2020) used survey data of 339,633 individuals linked with mortality data (2003–2007), and showed a similar effect estimate as Klompmaker et al. (2021), but with wider confidence intervals. Potentially these studies did not find significant associations because of an insufficient statistical power due to smaller study populations combined with shorter mortality follow-up periods.

Our study findings were consistent for different dementia subtypes. Two of the abovementioned studies additionally assessed dementia mortality (all dementia types, ICD-10 codes: F00-F03). Klompmaker et al. (2021) showed a significant 4% reduction in the risk of dementia mortality with increased surrounding greenness. Klompmaker et al. (2020) reported a similar but non-significant effect. Other longitudinal studies using health administrative databases found a beneficial association between green spaces and an outcome including all dementia types and AD (ICD-10 codes: F00-F03, G30, respectively) [19, 20]. However, studies evaluating the effect of green spaces on AD alone have shown contradictory findings [11–13, 21]. Moreover, we are not aware of prior studies evaluating the association between green spaces and vascular dementia. Thus, further research is needed to confirm our results.

Suggested mechanisms underlying the direct beneficial effect of exposure to green spaces on neurodegenerative disease mortality include inducing psychological restoration and reducing stress [8], potentially preventing depression, a risk factor for dementia [54]. Additionally, there is suggestive evidence that green spaces could increase physical activity in the older population [6], reducing the risk of dementia and AD [55]. Social isolation is moreover associated with an increased risk of cognitive decline and dementia [56], and greener neighbourhoods could enhance social cohesion and mitigate feelings of loneliness in older adults [57, 58]. Finally, green spaces could contribute to the mitigation of environmental hazards, including air pollution [59]. Our findings suggest that the associations between residential greenness and all neurodegenerative disease mortality are partly mediated by reductions in NO_2_ concentrations. Results for PM_2.5_ were similar but not significant. For AD, vascular dementia, and unspecified dementia mortality, we observed that the estimates of the indirect effects (ACME) and the direct effects (ADE) had opposite signs. This suggests inconsistent mediation; hence we considered the interpretation of these results inappropriate. So far, only one longitudinal study on green spaces and cognitive function explored potential mediation by air pollution, but no evidence of mediation effects was found [16]. Current evidence establishes a strong link between air pollution and AD and dementia [26]. The filtering effect of green spaces removing pollutants from the atmosphere has been proven to be generally small [60, 61]. However, green spaces could decrease temperature in cities [62], indirectly improving air quality by reducing the generation, transportation and toxicity of pollutants [63]. Likewise, fewer sources of air pollution are found in greener areas [40]. Noise pollution or proximity to roads have also been associated with an increased risk of dementia independently from air pollution levels [3, 4], but we unfortunately lacked such information.

As part of the sensitivity analyses, we used perceived neighbourhood greenness as alternative exposure indicator, where we found no association with AD nor vascular dementia mortality and a stronger association with unspecified dementia compared to the results of our main models using surrounding greenness. We suspect that perceived neighbourhood greenness may partly capture certain aspects of neighbourhood socioeconomic position (SEP). As such, this model may be overadjusted for SEP. Our findings are probably explained by individuals with high SEP having a higher likelihood of getting a record of dementia aetiology in death certificates [64], and, moreover, residing more often in areas where a higher proportion of individuals report very good neighbourhood greenspace provision.

No beneficial association between greenness and PD mortality was found, probably a result of the differing aetiology of PD compared to that of the other neurodegenerative diseases under study [65]. Additionally, in sensitivity analysis the associations with PD changed direction and became beneficial when commuting zone residents were excluded. Morphologically, these areas are characterized by an extensive land use in both housing and commercial activities [31]. Thus, we may speculate that increased risk of PD mortality could partly be explained by exposure to agricultural land, potentially encompassing exposure to pesticides, although this has been mainly explored for agricultural workers [65], being the evidence available for residential exposures currently limited [66]. Furthermore, we were not able to further explore this hypothesis since we lacked data on different types of green such as agricultural land.

Comparison between different population subgroups in the stratified analyses should be done with caution, given statistical restrictions in such interpretations. We observed strongest beneficial effects of living near greener areas in the lower educated for all neurodegenerative diseases and AD mortality. No clear patterns were found for other studied causes. Similarly, the longitudinal study of de Keijzer et al. (2018) did not find consistent evidence for differences between education groups in the association with residential greenness and cognitive decline [16]. Regarding neighbourhood SEP, we observed a general trend for all neurodegenerative disease and dementia mortality, where strongest associations with residential greenness were found in more socially deprived areas. The aforementioned study by de Keijzer et al. (2018) observed similar patterns with cognitive decline. Lower SEP has been linked to both poorer living conditions and limited access to resources which may be related to increased risk of cognitive decline in later life [67]. Additionally, availability of residential green has been associated to reduced risk of mortality, where health benefits seemed to be largest among most deprived population groups [68]. Such gradient was partly confirmed by our study results, although for AD mortality we found strongest beneficial associations in individuals residing in wealthier areas. This contradicts the findings from Brown et al. (2018), in which a trend by neighbourhood SEP in the association between greenness and AD prevalence was found, showing the strongest estimate in low-income neighbourhoods.

Our study comes with several limitations, one of which is the lack of available information on lifestyle factors potentially related to most neurodegenerative diseases, such as physical activity, smoking, or alcohol consumption [26]. Prior studies that were able to account for these factors observed a small attenuation in the association [22, 23]. However, the role of lifestyle factors in the associations under study is unclear, given that they are not directly associated with residential greenness. Physical activity could lie in the causal pathway between surrounding greenness and neurodegenerative diseases. Unfortunately, we did not have information on this specific lifestyle factor, and we could not test this hypothesized mechanism. However, we were able to test other suggested mechanism, i.e., reductions in air pollution concentrations. Furthermore, our study did not include time-varying variables of exposure throughout the follow-up period. We only had one measure of surrounding greenness for the year 2006, close to the middle of the follow-up period, which is another limitation of our study. We assumed that, although the quantity of green spaces may vary across time, their spatial distribution remains relatively stable. However, no other exposure information was available for other years to test this. Moreover, exposure assessment was based on the geocoded residential address at baseline (2001), and we lacked information on residential mobility during follow-up (2001–2014). Still, we were able to limit the analyses to a group of residents who did not move in the last 10 years prior to baseline, confirming main results. Additionally, we restricted our analyses to individuals who did not die or emigrate from the beginning of the study (1st of October 2001) until the year of measurement of the indicator surrounding greenness (1st of January 2006), and findings did not change considerably. Surrounding greenness captured all types and sizes of green spaces, independently of these being private or public. Also, limiting greenspace exposure assessment to the residence may result in exposure misclassification, as it potentially ignores exposure in other life spheres, e.g., working place. Similarly, episodes of nature interaction were not measured in quality (e.g., accessibility, type of use) nor in time (e.g., frequency, duration). We therefore acknowledge potential bias in our estimates, although its direction and magnitude remain unclear. We also relied on baseline information of sociodemographic characteristics, but these may not vary considerably among the older population. Applied missing values techniques present limitations given that the missing values may not be completely at random, which potentially affects generalizability of our findings. However, by using two approaches to handle missing data (i.e., multiple imputation and listwise deletion), we may assume that main conclusions are fairly robust. Lastly, we excluded the institutionalized population (i.e., care homes) to minimise selection bias, given that almost half of individuals diagnosed with neurodegenerative diseases live in institutions [69].

Notwithstanding the limitations described, our study counts with an important number of strengths. We analysed the association between surrounding greenness and neurodegenerative diseases over a longer follow-up period (13.25 years) than prior studies [22–24]. We also relied on a high resolution environmental database, linked to the geocoded residential address of each individual officially residing in the five largest Belgian urban areas at baseline [36]. Our study was also the first to conduct mediation analyses in the studied associations by air pollution concentrations using individually linked exposure data. Finally, using large administrative data allowed us to study effect modification by gender and socioeconomic characteristics through stratification for representative subgroups.

## Conclusions

In a large population-based cohort of older adults, we observed a reduced risk of all and specific neurodegenerative disease mortality (except for Parkinson’s disease) associated to exposure to residential surrounding greenness. Associations remained robust for all neurodegenerative disease mortality when accounting for air pollution, but not for the majority of specific mortality outcomes. Additionally, the beneficial effect of exposure to green spaces on neurodegenerative disease mortality might be stronger in lower educated groups and individuals residing in more deprived neighbourhoods. In Europe, neurological disorders represent the third leading cause of death and disability, after cardiovascular diseases and cancer [70]. Still, up to 40% of dementia cases can be potentially prevented [71]. Our results highlight the importance of the living environment to promote healthy ageing and reduce the burden of neurodegenerative diseases, especially among the most vulnerable populations. Future research is needed to confirm our findings in other settings and to explore other underlying mechanisms linking surrounding greenness with neurodegenerative diseases.

## Supplementary Information

**Additional file 1: Table S1.** Detailed description of the environmental indicators. Five largest Belgian urban areas, 2001-2014. **Table S2.** Stepwise adjustment of the associations (HR) and 95% confidence intervals (95%CI) between one IQR increment (0.22) of residential surrounding greenness (buffer size 500-m) and neurodegenerative disease mortality. Five largest Belgian urban areas, 2001-2014. **Table S3.** Associations (HR) and 95%CI of the associations between surrounding greenness (buffer size 500-m) categorised into quintiles of exposure and neurodegenerative disease mortality. Five largest Belgian urban areas, 2001-2014. **Table S4.** Computed average causal mediation effects (ACME) i.e., indirect effects, average direct effects (ADE) and proportion mediated, and their corresponding 95% confidence intervals (95%CI) of the association between surrounding greenness and specific outcomes of neurodegenerative disease mortality, potentially mediated by 2010 air pollution (PM_2.5_ and NO_2_) concentrations. Five largest Belgian urban areas, 2001-2014. **Table S5.** Stratified analyses of the number of events and the associations (HR and 95%CI) between IQR increments of residential greenness and neurodegenerative disease mortality, by gender, educational level, and neighbourhood SEP. Five largest Belgian urban areas, 2001-2014. **Table S6.** Sensitivity analyses of the associations (HR and 95%CI) between IQR increments of surrounding greenness and neurodegenerative disease mortality on the full (imputed) population (*n*=1,134,502). Five largest Belgian urban areas, 2001-2014. **Table S7.** Sensitivity analyses of the associations (HR and 95%CI) between IQR increments of surrounding greenness (buffer size: 500-m) and neurodegenerative disease mortality on population groups. Five largest Belgian urban areas, 2001-2014. **Figure S1.** Directed Acyclic Graph (DAG) of the main association. **Figure S2.** Linearity of the exposure-response relationship using natural splines with three degrees of freedom. *Note:* Cox proportional hazard models stratified by age group and gender, including a frailty term for urban area, and adjusted by migrant background, household living arrangement, educational level, housing tenure, and area-level SEP. p-values resulting from the LRT comparing this model to the main model. Five largest Belgian urban areas, 2001-2014. **Figure S3.** Spearman correlations between residential surrounding greenness, ambient air pollution and area-level median net taxable income.

## Data Availability

The data that support the findings of this study includes identifying information on participants and was used under license for the current study, and hence not publicly available. Data codebooks and syntaxes used for the statistical analyses are however available from the authors upon request.

## Abbreviations

NDVI: Normalised Difference Vegetation Index
IQR: Interquartile range
HR: Hazard ratio
95%CI: 95% confidence interval
PM_2.5_: Particulate matter with an aerodynamic matter smaller than 2.5 μm
NO_2_: Nitrogen dioxide
AD: Alzheimer’s disease
PD: Parkinson’s disease
SEP: Socioeconomic position
