# Supplementary material for: Long-term exposure to residential greenness and neurodegenerative disease mortality among older adults: a 13-year follow-up cohort study
Source: Environ Health. 2022 May 7;21:49. doi: 10.1186/s12940-022-00863-x (PMC9077872; doi:10.1186/s12940-022-00863-x)
Supplement: Supplementary file 1 — Additional file 1: Table S1. Detailed description of the environmental indicators. Five largest Belgian urban areas, 2001-2014. Table S2. Stepwise adjustment of the associations (HR) and 95% confidence intervals (95%CI) between one IQR increment (0.22) of residential surrounding greenness (buffer size 500-m) and neurodegenerative disease mortality. Five largest Belgian urban areas, 2001-2014. Table S3. Associations (HR) and 95%CI of the associations between surrounding greenness (buffer size 500-m) categorised into quintiles of exposure and neurodegenerative disease mortality. Five largest Belgian urban areas, 2001-2014. Table S4. Computed average causal mediation effects (ACME) i.e., indirect effects, average direct effects (ADE) and proportion mediated, and their corresponding 95% confidence intervals (95%CI) of the association between surrounding greenness and specific outcomes of neurodegenerative disease mortality, potentially mediated by 2010 air pollution (PM2.5 and NO2) concentrations. Five largest Belgian urban areas, 2001-2014. Table S5. Stratified analyses of the number of events and the associations (HR and 95%CI) between IQR increments of residential greenness and neurodegenerative disease mortality, by gender, educational level, and neighbourhood SEP. Five largest Belgian urban areas, 2001-2014. Table S6. Sensitivity analyses of the associations (HR and 95%CI) between IQR increments of surrounding greenness and neurodegenerative disease mortality on the full (imputed) population (n=1,134,502). Five largest Belgian urban areas, 2001-2014. Table S7. Sensitivity analyses of the associations (HR and 95%CI) between IQR increments of surrounding greenness (buffer size: 500-m) and neurodegenerative disease mortality on population groups. Five largest Belgian urban areas, 2001-2014. Figure S1. Directed Acyclic Graph (DAG) of the main association. Figure S2. Linearity of the exposure-response relationship using natural splines with three degrees of freedom. Not [file 12940_2022_863_MOESM1_ESM.docx]

**Long-term exposure to residential greenness and neurodegenerative disease mortality among older adults: a 13-year follow-up cohort study**

Lucía Rodriguez-Loureiro, Sylvie Gadeyne, Mariska Bauwelinck, Wouter Lefebvre, Charlotte Vanpoucke, Lidia Casas

**SUPPLEMENTARY MATERIAL**

Table of Contents

[SUPPLEMENTARY TABLES 2](#_Toc100764989)

[Table S1. Detailed description of the environmental indicators. Five largest Belgian urban areas, 2001-2014. 3](#_Toc100764990)

[Table S2. Stepwise adjustment of the associations (HR) and 95% confidence intervals (95%CI) between one IQR increment (0.22) of residential surrounding greenness (buffer size 500-m) and neurodegenerative disease mortality. Five largest Belgian urban areas, 2001-2014. 4](#_Toc100764991)

[Table S3. Associations (HR) and 95%CI of the associations between surrounding greenness (buffer size 500-m) categorised into quintiles of exposure and neurodegenerative disease mortality. Five largest Belgian urban areas, 2001-2014. 5](#_Toc100764992)

[Table S4. Computed average causal mediation effects (ACME) i.e., indirect effects, average direct effects (ADE) and proportion mediated, and their corresponding 95% confidence intervals (95%CI) of the association between surrounding greenness and specific outcomes of neurodegenerative disease mortality, potentially mediated by 2010 air pollution (PM_2.5_ and NO_2_) concentrations. Five largest Belgian urban areas, 2001-2014. 6](#_Toc100764993)

[Table S5. Stratified analyses of the number of events and the associations (HR and 95%CI) between IQR increments of residential greenness and neurodegenerative disease mortality, by gender, educational level, and neighbourhood SEP. Five largest Belgian urban areas, 2001-2014. 7](#_Toc100764994)

[Table S6. Sensitivity analyses of the associations (HR and 95%CI) between IQR increments of surrounding greenness and neurodegenerative disease mortality on the full (imputed) population (n=1,134,502). Five largest Belgian urban areas, 2001-2014. 8](#_Toc100764995)

[Table S7. Sensitivity analyses of the associations (HR and 95%CI) between IQR increments of surrounding greenness (buffer size: 500-m) and neurodegenerative disease mortality on population groups. Five largest Belgian urban areas, 2001-2014. 9](#_Toc100764996)

[SUPPLEMENTARY FIGURES 10](#_Toc100764997)

[Figure S1. Directed Acyclic Graph (DAG) of the main association. 11](#_Toc100764998)

[Figure S2. Linearity of the exposure-response relationship using natural splines with three degrees of freedom. 12](#_Toc100764999)

[Figure S3. Spearman correlations between residential surrounding greenness, ambient air pollution and area-level median net taxable income. 13](#_Toc100765000)

# SUPPLEMENTARY TABLES

## **Table S1.** Detailed description of the environmental indicators. Five largest Belgian urban areas, 2001-2014.

|  |  | **Median** | **Q1** | **Q3** | **IQR** | **Min** | **Max** |
| --- | --- | --- | --- | --- | --- | --- | --- |
| **Full (imputed) population, N=1,134,502** | |  |  |  |  |  |  |
|  | **Surrounding greenness** |  |  |  |  |  |  |
|  | NDVI, 300-m | 0.60 | 0.47 | 0.70 | 0.23 | 0.13 | 0.94 |
|  | NDVI, 500-m | 0.62 | 0.49 | 0.72 | 0.22 | 0.13 | 0.93 |
|  | NDVI, 1000-m | 0.64 | 0.52 | 0.74 | 0.22 | 0.16 | 0.91 |
|  | **Perceived neighbourhood greenness (%)** | 22.57 | 13.21 | 34.62 | 21.41 | 0.00 | 100.0 |
|  | **Air pollution, PM_2.5_ (µg/m^3^)** | 16.73 | 15.46 | 18.82 | 3.36 | 7.72 | 40.55 |
|  | **Air pollution, NO_2_ (µg/m^3^)** | 26.56 | 21.47 | 33.69 | 12.22 | 6.95 | 154.79 |
| **Complete case population, N=911,648** | |  |  |  |  |  |  |
|  | **Surrounding greenness** |  |  |  |  |  |  |
|  | NDVI, 300-m | 0.59 | 0.47 | 0.69 | 0.22 | 0.13 | 0.94 |
|  | NDVI, 500-m | 0.61 | 0.49 | 0.71 | 0.22 | 0.13 | 0.93 |
|  | NDVI, 1000-m | 0.64 | 0.52 | 0.74 | 0.22 | 0.17 | 0.91 |
|  | **Perceived neighbourhood greenness (%)** | 22.54 | 13.24 | 34.56 | 21.32 | 0.00 | 100.0 |
|  | **Air pollution, PM_2.5_ (µg/m^3^)** | 16.82 | 15.52 | 18.83 | 3.31 | 7.76 | 40.55 |
|  | **Air pollution, NO_2_ (µg/m^3^)** | 26.77 | 21.69 | 33.67 | 11.98 | 7.96 | 154.79 |

## **Table S2.** Stepwise adjustment of the associations (HR) and 95% confidence intervals (95%CI) between one IQR increment (0.22) of residential surrounding greenness (buffer size 500-m) and neurodegenerative disease mortality. Five largest Belgian urban areas, 2001-2014.

|  |  | **M1** | **M2** | **M3** |
| --- | --- | --- | --- | --- |
|  |  | **HR (95%CI)** | **HR (95%CI)** | **HR (95%CI)** |
| **Neurodegenerative disease mortality outcome** | |  |  |  |
|  | **All neurodegenerative diseases** | 0.956 (0.945, 0.967) | 0.942 (0.930, 0.953) | 0.959 (0.946, 0.973) |
|  | **Alzheimer's disease** | 0.960 (0.940, 0.980) | 0.935 (0.914, 0.956) | 0.952 (0.927, 0.977) |
|  | **Vascular dementia** | 0.909 (0.874, 0.946) | 0.918 (0.880, 0.958) | 0.948 (0.902, 0.997) |
|  | **Unspecified dementia** | 0.937 (0.921, 0.953) | 0.924 (0.907, 0.940) | 0.948 (0.928, 0.968) |
|  | **Parkinson's disease** | 1.032 (1.001, 1.064) | 1.010 (0.978, 1.043) | 1.007 (0.970, 1.046) |
| Note: HR, Hazard Ratio; 95%CI, 95% confidence intervals. Cox regression models using age as the underlying time scale for the follow-up period October 1, 2001 - December 31, 2014. Model 1 included the baseline hazard, the strata terms for age and gender and the frailty term for urban areas; Model 2 added migrant background, household composition, educational level, and housing tenure; and Model 3 included median net taxable income at the statistical sector level. | | | | |

## **Table S3.** Associations (HR) and 95%CI of the associations between surrounding greenness (buffer size 500-m) categorised into quintiles of exposure and neurodegenerative disease mortality. Five largest Belgian urban areas, 2001-2014.

|  | **Neurodege-nerative diseases** | **Alzheimer's disease** | **Vascular dementia** | **Unspecified dementia** | **Parkinson's disease** |
| --- | --- | --- | --- | --- | --- |
|  | **HR (95%CI)** | **HR (95%CI)** | **HR (95%CI)** | **HR (95%CI)** | **HR (95%CI)** |
| **Linear term [IQR increments]** | 0.959 (0.946, 0.973) | 0.952 (0.927, 0.977) | 0.948 (0.902, 0.997) | 0.948 (0.928, 0.968) | 1.007 (0.970, 1.046) |
| **Q1 (least green)** | 1.000 | 1.000 | 1.000 | 1.000 | 1.000 |
| **Q2** | 0.962 (0.940, 0.985) | 0.993 (0.950, 1.037) | 0.968 (0.893, 1.050) | 0.934 (0.902, 0.967) | 0.985 (0.924, 1.051) |
| **Q3** | 0.957 (0.933, 0.981) | 1.014 (0.969, 1.061) | 0.941 (0.863, 1.027) | 0.919 (0.886, 0.953) | 0.960 (0.897, 1.027) |
| **Q4** | 0.948 (0.923, 0.974) | 0.942 (0.896, 0.990) | 0.964 (0.877, 1.059) | 0.940 (0.903, 0.978) | 0.986 (0.918, 1.060) |
| **Q5 (most green)** | 0.918 (0.892, 0.944) | 0.879 (0.834, 0.927) | 0.917 (0.830, 1.013) | 0.911 (0.873, 0.950) | 0.996 (0.925, 1.073) |
| HR, Hazard Ratio; 95%CI, 95% Confidence Interval; IQR, Interquartile range. Cox proportional hazard models using age as the underlying time scale for the follow-up period 2001-2014. Main models (M3) stratified by gender and 5-year age groups, adjusted by migrant background, household living arrangement, educational level, housing tenure and median net taxable income at the level of the statistical sector, accounting for the cluster effects of the urban areas. Quintiles of exposure for surrounding greenness: Q1 (least green): [0.127,0.457]; Q2: (0.457,0.572]; Q3: (0.572,0.655]; Q4: (0.655,0.737]; Q5: (0.737,0.926]. | | | | | |

## **Table S4.** Computed average causal mediation effects (ACME) i.e., indirect effects, average direct effects (ADE) and proportion mediated, and their corresponding 95% confidence intervals (95%CI) of the association between surrounding greenness and specific outcomes of neurodegenerative disease mortality, potentially mediated by 2010 air pollution (PM_2.5_ and NO_2_) concentrations. Five largest Belgian urban areas, 2001-2014.

|  | **ACME (95%CI)** | **ADE (95%CI)** | **Proportion mediated (95%CI)** |
| --- | --- | --- | --- |
| **All neurodegenerative disease mortality** |  |  |  |
| **PM_2.5_ (µg/m^3^)** | 3.79 (-0.95, 8.51) | 12.95 (6.17, 19.61) | 0.23 (-0.06, 0.55) |
| **NO_2_** **(µg/m^3^)** | 6.46 (1.59, 11.36) | 10.24 (3.26, 17.07) | 0.39 (0.09, 0.76) |
| **Alzheimer's disease mortality** |  |  |  |
| **PM_2.5_ (µg/m^3^)** | 78.0 (44.18, 112.04) | -1.53 (-52.43, 46.17) | 1.01 (0.52, 2.10) |
| **NO_2_** **(µg/m^3^)** | 106.23 (72.56, 141.38) | -30.84 (-83.37, 19.23) | 1.40 (0.80, 2.83) |
| **Vascular dementia mortality** |  |  |  |
| **PM_2.5_ (µg/m^3^)** | 447.62 (155.32, 756.21) | -97.16 (-565.24, 315.41) | 1.21 (0.23, 6.66) |
| **NO_2_** **(µg/m^3^)** | 269.55 (-26.86, 572.11) | 80.99 (67.62, 102.45) | 0.73 (-0.35, 4.26) |
| **Unspecified dementia mortality** |  |  |  |
| **PM_2.5_ (µg/m^3^)** | -32.76 (-54.68, -10.83) | 98.79 (68.81, 127.52) | -0.50 (-0.98, -0.16) |
| **NO_2_** **(µg/m^3^)** | -30.13 (-53.01, -7.60) | 96.19 (64.91, 125.84) | -0.45 (-0.94, -0.11) |
| Models were adjusted for age, gender, urban area, migrant background, household living arrangement, educational level, housing tenure, and median net taxable income in the census tract. Results computed from the 25 imputed datasets. | | | |

## **Table S5.** Stratified analyses of the number of events and the associations (HR and 95%CI) between IQR increments of residential greenness and neurodegenerative disease mortality, by gender, educational level, and neighbourhood SEP. Five largest Belgian urban areas, 2001-2014.

|  | **Neurodegenerative diseases** | | **Alzheimer's disease** | | **Vascular dementia** | | **Unspecified dementia** | | **Parkinson's disease** | |
| --- | --- | --- | --- | --- | --- | --- | --- | --- | --- | --- |
|  | **n** | **HR (95%CI)** | **n** | **HR (95%CI)** | **n** | **HR (95%CI)** | **n** | **HR (95%CI)** | **n** | **HR (95%CI)** |
| **Gender** |  |  |  |  |  |  |  |  |  |  |
| Men | 26,741 | 0.960 (0.938,  0.982) | 7,368 | 0.967 (0.925,  1.010) | 2,501 | 0.931 (0.864,  1.004) | 10,925 | 0.947 (0.914,  0.981) | 5,425 | 0.990 (0.941,  1.042) |
| Women | 42,408 | 0.958 (0.941,  0.976) | 13,671 | 0.943 (0.913,  0.974) | 3,150 | 0.962 (0.900,  1.028) | 20,377 | 0.948 (0.923,  0.973) | 4,629 | 1.027 (0.972,  1.086) |
| **Educational level** | | | | | | | | | | |
| Tertiary education | 6,748 | 0.963 (0.917,  1.012) | 2,169 | 0.949 (0.874,  1.030) | 526 | 0.963 (0.810,  1.145) | 2,473 | 0.955 (0.880,  1.036) | 1,302 | 0.981 (0.884,  1.087) |
| Higher secondary education | 10,060 | 0.999 (0.960,  1.040) | 3,141 | 0.957 (0.890,  1.028) | 747 | 0.946 (0.816,  1.096) | 4,324 | 1.014 (0.954,  1.078) | 1,608 | 0.987 (0.896,  1.088) |
| Lower secondary education | 17,873 | 0.968 (0.939,  0.996) | 5,618 | 0.995 (0.943,  1.049) | 1,431 | 0.930 (0.836,  1.035) | 7,791 | 0.944 (0.902,  0.989) | 2,698 | 1.016 (0.943,  1.095) |
| Primary/No formal education | 34,468 | 0.954 (0.934,  0.974) | 10,111 | 0.934 (0.898,  0.970) | 2,947 | 0.956 (0.889,  1.028) | 16,714 | 0.935 (0.907,  0.964) | 4,446 | 1.042 (0.982,  1.105) |
| **Median net taxable income (statistical sector)** | | | | | | | | | | |
| Q4 (least deprived) | 15,384 | 0.974 (0.936,  1.013) | 4,379 | 0.906 (0.840,  0.976) | 1,261 | 1.043 (0.908,  1.198) | 6,915 | 1.001 (0.944,  1.062) | 2,481 | 0.975 (0.883,  1.077) |
| Q3 | 17,169 | 0.957 (0.930,  0.985) | 4,841 | 0.932 (0.880,  0.987) | 1,425 | 0.870 (0.784,  0.966) | 8,080 | 0.973 (0.931,  1.016) | 2,528 | 0.929 (0.860,  1.004) |
| Q2 | 18,262 | 0.961 (0.936,  0.987) | 5,627 | 0.983 (0.936,  1.032) | 1,468 | 1.007 (0.916,  1.106) | 8,239 | 0.912 (0.876,  0.949) | 2,621 | 1.072 (1.001,  1.148) |
| Q1 (most deprived) | 18,334 | 0.948 (0.926,  0.971) | 6,192 | 0.948 (0.910,  0.988) | 1,497 | 0.881 (0.811,  0.957) | 8,068 | 0.930 (0.898,  0.964) | 2,424 | 1.073 (1.007,  1.143) |
| HR, Hazard Ratio; 95%CI, 95% Confidence Interval; Quartiles of exposure of area median net taxable income: Q1 (5,676-16,471], Q2 (16,471-19,094], Q3 (19,094-21,091], Q4 (21,091-51,473]. Cox proportional hazard models using age as the underlying time scale for the follow-up period 2001-2014. Models stratified by gender and 5-year age groups, adjusted by migrant background, household living arrangement, educational level, housing tenure and median net taxable income at the level of the statistical sector, and including a frailty term for the urban areas. | | | | | | | | | | |

## **Table S6.** Sensitivity analyses of the associations (HR and 95%CI) between IQR increments of surrounding greenness and neurodegenerative disease mortality on the full (imputed) population (n=1,134,502). Five largest Belgian urban areas, 2001-2014.

|  | **Neurodegenerative diseases** | **Alzheimer's disease** | **Vascular dementia** | **Unspecified dementia** | **Parkinson's disease** |
| --- | --- | --- | --- | --- | --- |
|  | **HR (95%CI)** | **HR (95%CI)** | **HR (95%CI)** | **HR (95%CI)** | **HR (95%CI)** |
| **Main models** | 0.959 (0.946, 0.973) | 0.952 (0.927, 0.977) | 0.948 (0.902, 0.997) | 0.948 (0.928, 0.968) | 1.007 (0.970, 1.046) |
| **1. Different buffers of surrounding greenness** |  |  |  |  |  |
| Buffer size 300-m | 0.962 (0.948, 0.975) | 0.958 (0.933, 0.983) | 0.945 (0.900, 0.993) | 0.947 (0.927, 0.967) | 1.016 (0.979, 1.055) |
| Buffer size 1,000-m | 0.954 (0.940, 0.967) | 0.936 (0.911, 0.961) | 0.941 (0.895, 0.989) | 0.952 (0.932, 0.972) | 0.999 (0.962, 1.038) |
| **2. Underlying cause of death** | 0.970 (0.951, 0.988) | 0.978 (0.946, 1.012) | 0.939 (0.874, 1.009) | 0.942 (0.914, 0.970) | 1.053 (0.998, 1.112) |
| **3. Perceived neighbourhood greenness** | 0.981 (0.970, 0.992) | 1.034 (1.013, 1.055) | 1.001 (0.963, 1.041) | 0.925 (0.910, 0.941) | 1.024 (0.994, 1.055) |
| **4. Adjustment for unemployment rate (alternative neighbourhood SEP indicator)** | 0.962 (0.948, 0.976) | 0.985 (0.959, 1.011) | 0.939 (0.893, 0.987) | 0.930 (0.910, 0.950) | 1.016 (0.977, 1.055) |
| HR, Hazard Ratio; 95%CI, 95% Confidence Interval. Cox proportional hazard models using age as the underlying time scale for the follow-up period 2001-2014. Models stratified by gender and 5-year age groups, adjusted by migrant background, household living arrangement, educational level, housing tenure and median net taxable income at the level of the statistical sector, accounting for the cluster effects of the urban areas. | | | | | |

## **Table S7.** Sensitivity analyses of the associations (HR and 95%CI) between IQR increments of surrounding greenness (buffer size: 500-m) and neurodegenerative disease mortality on population groups. Five largest Belgian urban areas, 2001-2014.

|  | **Population size (n)** | **Neurodegenerative diseases** | | **Alzheimer's disease** | | **Vascular dementia** | | **Unspecified dementia** | | **Parkinson's disease** | |
| --- | --- | --- | --- | --- | --- | --- | --- | --- | --- | --- | --- |
|  |  | **n** | **HR (95%CI)** | **n** | **HR (95%CI)** | **n** | **HR (95%CI)** | **n** | **HR (95%CI)** | **n** | **HR (95%CI)** |
| **Main models** | 1,134,502 | 69,149 | 0.959 (0.946,  0.973) | 21,039 | 0.952 (0.927,  0.977) | 5,651 | 0.948 (0.902,  0.997) | 31,302 | 0.948 (0.928,  0.968) | 10,054 | 1.007 (0.970,  1.046) |
| **5. Population groups** |  |  |  |  |  |  |  |  |  |  |  |
| a.  Complete case population | 911,648 | 52,780 | 0.973 (0.958,  0.989) | 16,161 | 0.963 (0.935,  0.992) | 4,204 | 0.978 (0.924,  1.036) | 23,570 | 0.963 (0.940,  0.986) | 7,932 | 1.009 (0.968,  1.052) |
| b. Non-movers | 1,003,052 | 62,247 | 0.960 (0.945,  0.974) | 18,872 | 0.947 (0.922,  0.974) | 5,046 | 0.947 (0.899,  0.999) | 28,321 | 0.941 (0.921,  0.962) | 9,016 | 1.010 (0.971,  1.052) |
| c. Individuals with at least 4.25 years of follow-up | 957,688 | 52,261 | 0.962 (0.946,  0.978) | 16,607 | 0.956 (0.928  0.985) | 3,870 | 0.966 (0.910,  1.026) | 23,789 | 0.951 (0.928,  0.974) | 7,345 | 1.008 (0.965,  1.054) |
| d. Belgians | 1,001,938 | 62,816 | 0.954 (0.940,  0.969) | 18,751 | 0.940 (0.914,  0.966) | 5,122 | 0.941 (0.893,  0.991) | 28,721 | 0.941 (0.921,  0.962) | 9,195 | 0.998 (0.960,  1.039) |
| e. Aged 60-80 | 957,984 | 44,922 | 0.980 (0.962,  0.997) | 14,561 | 0.971 (0.941,  1.003) | 3,331 | 0.994 (0.932,  1.061) | 18,280 | 0.961 (0.935,  0.988) | 7,559 | 1.002 (0.959,  1.047) |
| f. City centre | 653,973 | 41,477 | 0.971 (0.952,  0.991) | 13,116 | 0.985 (0.950,  1.022) | 3,487 | 0.993 (0.926,  1.065) | 18,466 | 0.952 (0.924,  0.981) | 5,814 | 0.962 (0.911,  1.016) |
| HR, Hazard Ratio; 95%CI, 95% Confidence Interval. Cox proportional hazard models using age as the underlying time scale for the follow-up period 2001-2014. Models stratified by gender and 5-year age groups, adjusted by migrant background, household living arrangement, educational level, housing tenure and median net taxable income at the level of the statistical sector, accounting for the cluster effects of the urban areas. | | | | | | | | | | | |

# SUPPLEMENTARY FIGURES

## **Figure S1.** Directed Acyclic Graph (DAG) of the main association.

## **Figure S2.** Linearity of the exposure-response relationship using natural splines with three degrees of freedom.

*Note:* Cox proportional hazard models stratified by age group and gender, including a frailty term for urban area, and adjusted by migrant background, household living arrangement, educational level, housing tenure, and area-level SEP. p-values resulting from the LRT comparing this model to the main model. Five largest Belgian urban areas, 2001-2014.

## **Figure S3.** Spearman correlations between residential surrounding greenness, ambient air pollution and area-level median net taxable income.
